# Materials, Mechanics, and Patterning Techniques for Elastomer-Based Stretchable Conductors

**DOI:** 10.3390/mi8010007

**Published:** 2016-12-27

**Authors:** Xiaowei Yu, Bikram K. Mahajan, Wan Shou, Heng Pan

**Affiliations:** Department of Mechanical and Aerospace Engineering, Missouri University of Science and Technology, Rolla, MO 65401, USA; xy5w8@mst.edu (X.Y.); bkm6g7@mst.edu (B.K.M.); ws9n5@mst.edu (W.S.)

**Keywords:** stretchable conductors, elastomers, patterning techniques, direct printing, transfer printing

## Abstract

Stretchable electronics represent a new generation of electronics that utilize soft, deformable elastomers as the substrate or matrix instead of the traditional rigid printed circuit boards. As the most essential component of stretchable electronics, the conductors should meet the requirements for both high conductivity and the capability to maintain conductive under large deformations such as bending, twisting, stretching, and compressing. This review summarizes recent progresses in various aspects of this fascinating and challenging area, including materials for supporting elastomers and electrical conductors, unique designs and stretching mechanics, and the subtractive and additive patterning techniques. The applications are discussed along with functional devices based on these conductors. Finally, the review is concluded with the current limitations, challenges, and future directions of stretchable conductors.

## 1. Introduction

Since the pioneering work on buckling phenomena in metallic film/elastomer composite [1], researchers started to pay attentions to the field of “stretchable electronics”. Stretchable electronics represent a new generation of electronics that utilize soft, deformable elastomers as the substrate or matrix instead of rigid printed circuit boards. Unlike “flexible electronics” which utilize thin plastic substrates to endow the devices with conformity to bending and twisting [2,3], “stretchable electronics” are fascinating and challenging because of their capability to stretch and compress over a large scale, in addition to conformability [4,5,6,7,8,9,10,11].

Materials have been playing an important role in the development of stretchable conductors in the past two decades. Due to their high stretchability, elastomeric materials are commonly chosen as the supporting substrate or matrix for stretchable conductors and integrated device systems. Along with the traditional metallic conductors [1,12,13,14], several research groups have focused on developing conductive nanomaterials, including silver nanowires (AgNWs) [15], carbon nanotubes (CNTs) [16], etc. A substantial amount of work has been done to combine the rigid, brittle conductors with the flexible and soft elastomers, and overcoming the mismatches between their elastic behaviors. Unique designs and stretching mechanics have been proposed to harmonize the mismatches and integrate materials with widely different properties as one unique system. Patterning the conductors is one of the key techniques for the successful fabrication of stretchable electronic devices. The common patterning techniques for stretchable conductors include lithography [17,18], screen/stencil printing [19,20,21], direct printing [14,22,23,24,25,26,27,28,29,30,31], and transfer printing [32,33,34]. The patterning methods used in wafer-based electronics have also been adopted in stretchable electronics. Besides that, direct printing is emerging as an alternative to the conventional subtractive patterning method, with the recent development of additive manufacturing.

Despite their short history and limited scalability in manufacturing, it is quite clear that stretchable electronic devices would have a huge impact on future consumer electronics [4]. Because of their soft and conformable nature, stretchable electronics have shown great potential in biomedical engineering, e.g., epidermal electronic devices [18,35] and implantable devices [36,37]. Many studies have demonstrated the application of stretchable conductors in various devices and integrated systems, with functions such as sensing, display, and energy storage and conversion.

In this review, we focus on the basic building block of stretchable electronics, the elastomer-based stretchable conductors. The materials for elastomeric substrates and electrical conductors, and the mechanics of various types of stretchable conductors, are summarized in Section 2 and Section 3. Section 4 reviews the patterning techniques used in stretchable conductors, followed by a brief discussion on their applications in Section 5. Finally, in Section 6, current limitations, challenges, and the future directions of stretchable conductors are discussed.

## 2. Materials

It has been recognized that there are two basic approaches to making stretchable conductors: to exploit structures that are stretchable and materials that are stretchable [4,8,10,38,39]. The first approach involves designing commonly used conductors into particular structures that are stretchable. The latter employs a simple design, but utilizes materials that are stretchable, such as carbon nanotubes, metallic nanowires, liquid metals, and ionic liquids. Some studies combine these two approaches to achieve enhanced stretchability [40,41,42,43,44,45,46,47].

In this section, the materials for elastomeric substrate and electrical conductors are summarized. The preparation, modification, and performance of the stretchable conductive materials are discussed. Along with these materials, the traditional metallic conductors are discussed, with an emphasis on the recently developed additive manufacturing methods.

### 2.1. Elastomers as the Substrate or Supporting Matrix

Elastomers are the fundamental supporting materials for stretchable conductors. They serve either as the matrix for conductive fillers and networks [48,49,50], or the substrate for the conductive films, tracks, and functional devices [13,18,51]. Elastomers not only provide the stretchability when the whole system is under strain, but also protect the devices and interconnects from large-scale deformation [12,52,53,54] and corrosive environments [55]. In epidermal electronics, elastomers facilitate the conformal contact of human skin with the sensing electrodes, and prevent the unnecessary contact with other electrical components [18,35,56]. Besides that, elastomers can also be used as functional dielectric materials [20,57,58,59].

The most important characteristic of the elastomers is their elasticity. Elastomers can easily sustain repetitive strain (typically larger than 100%), for thousands of stretch/release cycles. Desirable properties also include optical transparency, which facilitates optical applications in optoelectronics, photodetectors, light-emitting devices, solar cells, etc. [8,10]. They also have good biocompatibility, which makes the elastomers suitable for biomedical applications [60,61]. Elastomers typically have a high thermal expansion coefficient and have an inherent tendency of swelling in common solvents, [62] which can result in device failures [63,64]. However, thermal expansion and induced deformation can be exploited to make 3D buckling structures [1], which is one of the most common approaches to fabricating stretchable conductors. Swelling can also be cleverly utilized to introduce wrinkling patterns on the substrate surface [65,66].

The most extensively used elastomer is polydimethylsiloxane (PDMS), a commercially available silicone-based elastomer. [55]. PDMS is an ideal choice for a stretchable substrate/matrix as it is low-cost, transparent, biocompatible, easy to process, and permeable to air [67]. It is also known to have surface hydrophobicity, which can be modified to hydrophilicity by O_2_ plasma [68,69], UV/ozone [24,70,71], and chemical treatment [68]. However, after exposure to air for a certain time, the surface reverts back to its hydrophobic nature. Such property has been utilized to modify the surface stiffness (Young’s modulus) of PDMS [66,70,72], and to control the interfacial adhesion of PDMS with conductive materials [24,69]. Besides acting as the substrate/matrix, PDMS has been widely used in soft lithography [73], as an intermediate substrate (stamp) to transfer functional materials from the donor substrate to the target substrate.

**Figure 1:**
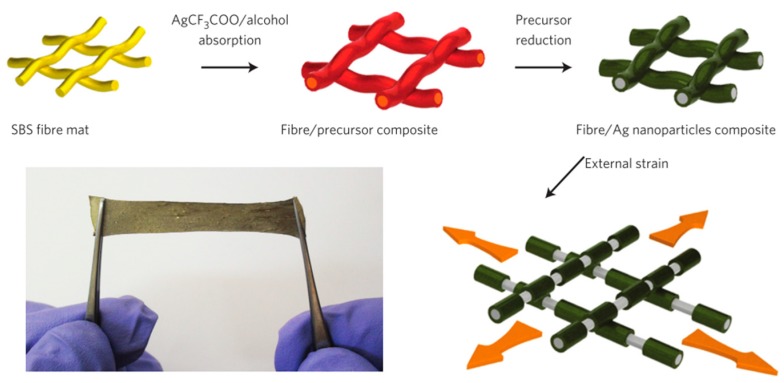
The schematic of fabrication process and the photograph of an SBS (styrene-block-butadiene-block-styrene) fiber mat-based stretchable conductor. Reprinted by permission from Macmillan Publishers Ltd.: *Nat. Nanotechnol.* [79], Copyright 2012.

Along with PDMS, some other elastomers are also employed as the substrate/matrix for various purposes. For example, Ecoflex [40,74,75] and polyurethane (PU) [49,76,77] are used in systems that require stretchability beyond the range of PDMS. Poly(isobutylene-co-isoprene) (IIR) is suitable for air- or moisture-sensitive applications [55] as it has a good gas diffusion barrier. Some rubbers, like polyolefin (POE) and poly (styrene-block-butadiene-block-styrene) (SBS), can be made into elastic fiber mats, taking advantage of their spinnability. These fiber mats have better stretchability than bulk elastomer blocks due to the network structure [78,79]. SBS fiber mat substrate is shown in Figure 1 as an example. Other reported elastomers include Dragon Skin [80], Solaris [81], acrylic elastomer [82,83,84,85], and nitrile butadiene rubber (NBR) [86]. Their elastic properties are summarized in Table 1.

### 2.2. Electrical Conductors

The conductive materials for stretchable conductors have more diversity compared to the elastomeric materials. They vary from inorganic to organic materials, solids to liquids, and bulk films to nano-scale percolation systems. In this section, the commonly used electrical conductors are introduced, including bulk metal films, metallic nanowires, carbon-based nanomaterials, conductive polymers, liquid metals, and ionic liquids. Bulk metal films have almost the same performance as the regular metallic conductors, with the exception that they are deformable owing to their ultra-small thickness (typically smaller than 1 μm) [12]. They have been extensively studied [1,12,13,92,93] and incorporated into various stretchable devices [4,35,94,95,96]. Recently, additive manufacturing has brought the bulk metal films and tracks to the research scope as materials for maskless, printable conductors [97,98,99]. Comparatively, conductive nanomaterials are relatively new, but their development has opened up a whole new area for stretchable conductors. Conductive nanomaterials include carbon-based nanomaterials and metallic nanowires. They can be incorporated into elastomeric materials by direct blending, or by fine architectures. Compared to metallic materials, polymeric materials have better intrinsic flexibility. The discovery of conductive polymers has brought another promising option for stretchable conductors. Liquid conductors, including liquid metals and ionic liquids, are advantageous in mobility in comparison to solid conductors. They can easily fit into the shape defined by the channel and have repeatable conductivity without forming any crack.

#### 2.2.1. Bulk Metal Films and Tracks

**Figure 2:**
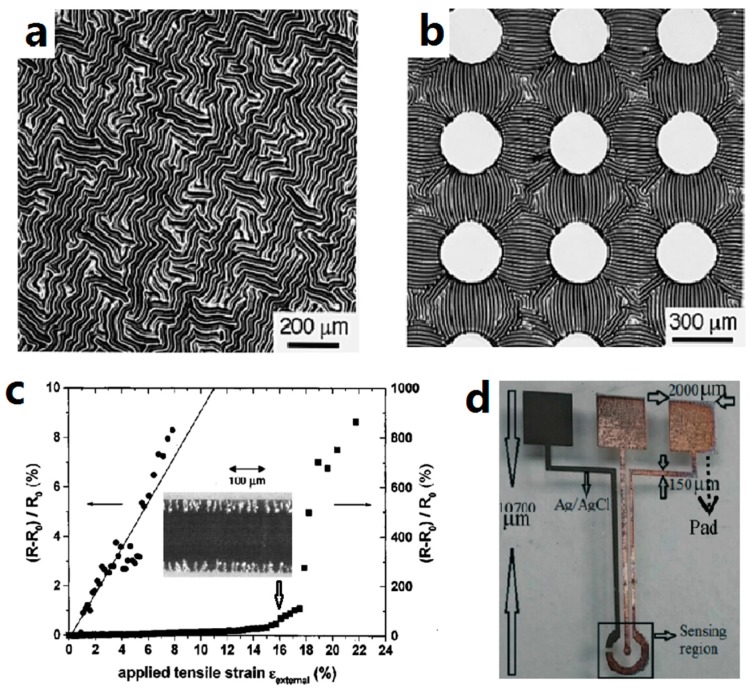
(**a**) Disordered buckling of Au film on flat, unconstraint PDMS (polydimethylsiloxane); (**b**) Ordered buckling of Au film on PDMS with flat circles (150 μm in radius) elevated by 10–20 μm; (**a**,**b**) are reprinted by permission from Macmillan Publishers Ltd.: *Nature* [1], Copyright 1998; (**c**) Normalized change in resistance of the Au stripe on PDMS under tensile strain. The left curve represents the linear behavior when the strain is below 8%. The inset is a photograph of the stripe stretched to 16.4%. Adapted from [12], with permission of AIP Publishing; (**d**) Ink-jet-printed Ag three-electrode electrochemical sensor. Reproduced from [25] with permission of The Royal Society of Chemistry.

Bulk metal films were studied extensively at the initial stages of development of stretchable conductors. It was found by Whitesides and his colleagues that disordered and ordered buckling (Figure 2a,b) occurred as the thin gold film evaporated on PDMS due to thermal contraction [1,72]. Researchers utilized this phenomenon to make stretchable gold stripes, which can be stretched up to 22%, as seen in Figure 2c [12]. Shortly after that, the lithographic method was incorporated and sinuous patterned gold tracks encapsulated in PDMS were fabricated, with a stretchability of 54% [13]. Gold was initially used because it is the most ductile metal. Later, emerging serpentine geometry designs were proposed to reduce strain in the metal [100,101,102,103]. Copper turned out to be the preferred material for serpentine-shape conductors due to lower cost and comparable conductivity.

With the development of additive manufacturing, direct writing techniques are showing up as an alternative to the traditional subtractive patterning methods. In the direct printing method, nanoparticle (NP) or metal-organic decomposition (MOD) inks are used, which are deposited from a printing head or nozzle to the substrate [98]. NP inks are usually a suspension of metallic nanoparticles dispersed in solvent. Silver NP inks are primarily used due to their high conductivity, good solvent-stability, air-inertness, and accessibility. MOD inks contain metal precursor and need additional steps (i.e., reduction) to generate metallic materials in-situ. In addition, sintering is required for both types of inks to evaporate solvent, decompose organic additives, and solidify the metallic particles. Figure 2d shows an optical image of an electrochemical sensor fabricated by ink-jet printing silver NP ink. The details of printing methods are introduced in Section 4.3.

#### 2.2.2. Metallic Nanowires

**Figure 3:**
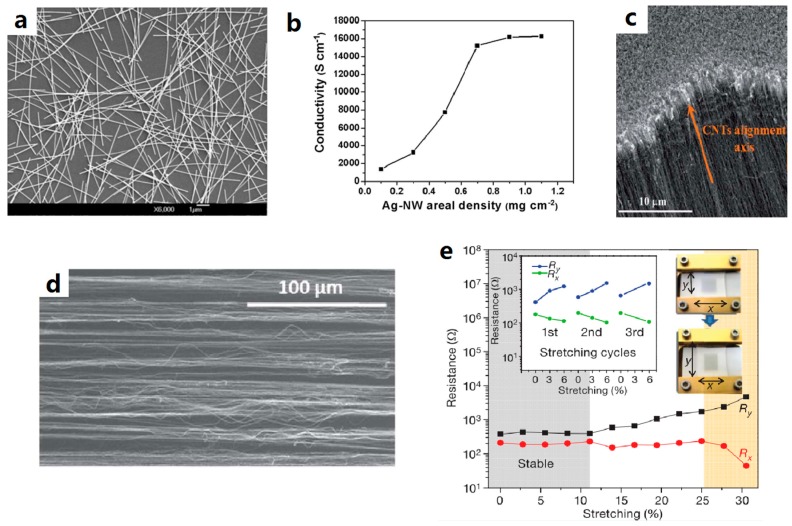
(**a**) SEM image of an AgNW (silver nanowire) network on glass [82]. ^©^ IOP Publishing. Reproduced with permission. All rights reserved; (**b**) Conductivity of an AgNW/PDMS conductor as a function of the areal density of AgNW. Reused from [113], under the terms of CC-BY license; (**c**) SEM image of vertically aligned CNT (carbon nanotube) forests. Reproduced from [130] with permission of The Royal Society of Chemistry; (**d**) SEM image of CNT ribbons drawn from a vertically aligned CNT forest. Reproduced from [131] with permission of The Royal Society of Chemistry; (**e**) Resistance of a graphene film transferred to a pre-stretched PDMS substrate isotopically stretched by 12%. The left inset shows the case in which the graphene film is transferred to an unstretched PDMS substrate. The right insets are photographs of the graphene film under vertical and horizontal strain. Reprinted by permission from Macmillan Publishers Ltd.: *Nature* [118], Copyright 2009.

Despite the high conductivity of bulk metal films and tracks, their utilization in stretchable electronics has to rely on special designs based on stretching mechanics due to their rigid and brittle nature. On the other hand, metallic nanowires are typically arranged into networks (shown in Figure 3a) where the nanowires with high aspect ratio are connected to each other and form the conductive paths. The intrinsic stretchability of the networks makes metallic nanowires an ideal candidate for stretchable conductors.

Silver nanowires (AgNWs) [20,40,59,82,104], copper nanowires (CuNWs) [85,105], and gold nanowires (AuNWs) [106,107] were reported serving as the conductors in stretchable electronics. AuNWs are costly, while CuNWs are easily oxidized in air [105]. Comparatively, AgNWs turned out to be the most popular choice because of their high conductivity and the well-established synthesis methods [108].

AgNWs were largely studied for transparent conductors [109,110] and were not incorporated into stretchable conductors until about five years ago [15,40,104]. AgNWs networks are usually fabricated by solution processes such as drop casting [20,59,82,104,111], vacuum filtration [40], and spray deposition [112,113]. Because elastomers are at risk to be attacked by the solvent and heat, AgNWs are typically solution-processed on glass or silicon, and then transferred to elastomeric substrate after evaporating the solvent. The performance of the AgNW-based stretchable conductors is related to various factors, such as the aspect ratio of AgNWs [40], the loading density of AgNWs [82,113], and interfacial adhesion between AgNWs and the substrates [82,112]. A short AgNW percolation network fabricated on 50% pre-stretched Ecoflex failed at 60% strain, while a long AgNW percolation network was able to retain conductivity with a strain of more than 80% [40]. Figure 3b shows the conductivity of an AgNW/PDMS conductor as a function of the areal density of AgNW, in which the conductivity increases with the loading density until the percolation threshold is reached [113]. The bonding between polyacrylate substrate and AgNWs was enhanced by introducing acrylic acid (AA) into the polymerization reaction. The sheet resistance of the AgNW/polyacrylate composite without AA was found to be 26% higher than that with AA [112].

Despite the high conductivity of the nanowires, the significant junction resistance makes the actual resistance of the whole nanowire network higher than expected [6]. The junction resistance can be effectively reduced by soldering. Thermal annealing, an effective method to get the junctions fused, was reported to reduce the sheet resistance of an AgNW network by one third [114]. Soldering with secondary functional materials or networks [74,115,116] would further improve the performance of the AgNW-based conductors under large tensile deformation. There are reports where AgNW networks were soldered by silver nanoparticles [116] and graphene oxides [74]. The conductor made by the former approach exhibited no obvious change in electrical conductivity, with strain up to 120%, and that by the latter was stretched to greater than 100% strain without losing electrical conductivity.

#### 2.2.3. Carbon-Based Nanomaterials

Carbon-based nanomaterials are a burning topic in materials science due to their unusual structures, which endow these materials with excellent mechanical and electrical properties. The application of the carbon-based nanomaterials in stretchable conductors is dated back from 1982 [117], where carbon black particles were dispersed in a silicon rubber matrix to make conductive elastomers. With the progress in materials research, carbon nanotubes [77] and graphene [118] were subsequently introduced to stretchable conductors. However, carbon-based nanomaterials offer lower electrical conductivity compared to metallic materials [8]. The simplest way of increasing the conductivity is to increase the loading density of the carbon-based nanomaterials, at the cost of sacrificing some of the mechanical properties and the transparency. High loading density would also make it difficult to obtain a uniform dispersion in solvents. Hence, there is always a trade-off between electrical performance and stretchability, transparency, and ease of processing. However, researchers have found clever ways to bypass this challenge and obtain good conductivity as well as good stretchability.

Dispersing carbon black [50,117,119] or graphite [120] in the elastomer matrix is a simple but effective method to fabricate stretchable conductors. Such composites have relatively low conductivity, which is desirable for micro-heaters [50]. Single-wall carbon nanotubes (SWCNTs) were reported to be made into composite dispersion and aerogel. The elastic conductor made from the dispersion of SWCNT, ionic liquid, and fluorinated copolymer retained good conductivity up to 134% strain [121]. The resistivity of SWCNT aerogels was found to increase only slightly (14%) at a strain of 100% [122]. Multi-wall carbon nanotubes (MWCNTs) can be dispersed in elastomer using a similar method to carbon blacks. The MWCNT/elastomer composites were reported to have percolation concentration ranging from 0.2–5 wt % [48].

Besides direct dispersion, many designs and processing techniques to assemble CNTs into stretchable conductors have been proposed. The vertically grown MWCNTs were embedded into polyurethane, retaining their conductivity when stretched up to 300% strain [76], and into PDMS for a load-bearing antenna [123]. CNT ribbons drawn from vertically grown CNT forest [124] were embedded into PDMS and stretched to 120% strain. Figure 3c,d show the SEM images of the vertically aligned MWCNT forest and the CNT ribbons, respectively. The CNT ribbons were fabricated into “out-of-plane” and “lateral” buckling architectures, which further enhanced the stretchability [42,43]. Cross-stacked super-aligned CNT film possessed a high intrinsic tensile strain of more than 35%, which was further improved by embedding the film into PDMS [125]. In addition, gap-island networks made by the overlapping SWCNT films [41], spring-like CNT ropes [126], hierarchical reticulate SWCNT architecture [44], etc. were demonstrated for stretchable, transparent, and highly conductive carbon-based conductors.

Graphene is a two-dimensional material with hexagonal honeycomb architecture and boasts of very high conductivity, stretchability, and transparency [46,47]. Successful synthesis of graphene in a large scale by chemical vapor deposition facilitated its application in stretchable conductors [118,127]. Graphene film transferred to a pre-stretched PDMS substrate was able to sustain isotopic strain up to 12% without change in resistance, as seen in Figure 3e [118]. Graphene was further patterned into structures such as meshes [47], ribbons [46,128], and serpentine tracks [27,129] for better stretchability by using pre-designed metallic templates or photolithography.

#### 2.2.4. Conductive Polymers

Compared to metals, polymers have better intrinsic flexibility. The discovery of conductive polymers has brought another promising option for stretchable conductors. Conductive polymers like polyaniline (PAni) [132], polypyrrole [133,134], poly(3,4-ethylenediox-ythiophene) (PEDOT) [49], and poly(3,4-ethylenediox-ythiophene):poly(styrene sulfonate) (PEDOT:PSS) [27,51,71] have been used in stretchable conductors despite some limitations such as environmental instability [135] and low stretchability [49,136]. PEDOT:PSS receives most of the attention among the conductive polymers because of its good conductivity and ease of processing. However, the breaking strain of a pristine PEDOT:PSS thin film is below 10% [136,137]. The stretchability of PEDOT:PSS film was improved by adding dimethylsulfoxide (DMSO) and Zonyl fluorosurfactant with the maximum strain of more than 20% [137]. The Zonyl modified PEDOT:PSS film on PDMS was found to form a 3D wavy buckling structure which promotes stretchability. The resistance of the film increased by a factor of only two at 50% strain [71]. Recently, a strain-insensitive PEDOT:PSS/acrylamide organogel was reported, which was stretched to more than 350% strain while it retained invariant resistance up to 50% strain [138]. Besides that, elastomeric conducting polyaniline networks with maximum elongation up to 90% ± 10% were fabricated by synthesizing polyaniline via molecular templates [139].

#### 2.2.5. Liquid Metals and Ionic Liquids

The most common liquid metal used in stretchable conductors is eutectic gallium indium alloy (EGaIn, with 75.5% Ga and 24.5% In). The advantages of EGaIn include mobility, self-healability [140], and processability at room temperature [141]. Typically, it is injected into microchannels fabricated by molding elastomers [53]. Some other methods, such as microcontact printing, stencil printing [142], and photolithography [143,144], are also used to fabricate stretchable conductors based on liquid metals.

Ionic liquids are molten salts that have conductivity comparable to many organic electrolyte solutions at room-temperature and are stable up to 300–400 °C [145]. Ma et al. reported the integration of ionic liquids with various supporting materials, such as cotton fabric, rubber film, rubber band, and sponge, which exhibited exceptional performance with high conductivity at strain greater than 600% [78].

## 3. Mechanics of the Stretchable Conductive Structure

As introduced in last section, elastomers serve as the stretchable substrate or matrix for stretchable conductors, while conductive materials provide the electrical functionalities. However, they have huge differences in elastic properties. Substantial effort has been made to combine the rigid, brittle conductors with the flexible and soft elastomers. From the aspect of the mechanics, many fancy structures and clever designs were proposed to harmonize the mismatches and integrate materials with widely different properties as a whole system.

### 3.1. Blending

Blending is a pure mechanical process which is simple and cost-effective. Several material, like carbon blacks [50,117,119], silver particles/flakes [19,50,86,146,147], MWCNTs [48,77], and PEDOTs [49] were reported as blending into elastomers. Such composites can be analyzed by the percolation theory, where the electrical conductivity is determined by the concentration of the fillers [86]:

σ = σ_0_(*V_f_* − *V_c_*)*^s^* (1)

where σ is the electrical conductivity, σ_0_ is the conductivity of the conductive filler, *V_f_* is the volumetric fraction of the filler, *V_c_* is the volumetric fraction at the percolation threshold, and *s* is the fitting exponent. The percolation threshold is the key to control the electrical conductivity of composites. Table 2 gives the percolation thresholds of the conductive fillers in elastomer matrix (in weight percent) for comparison. As seen in the table, the percolation threshold has a strong dependence on the size and shape of the materials. Compared to the particle fillers, MWCNT (as a 1D nanomaterial), has lower percolation threshold due to its high aspect ratio, and thus is the ideal candidate for transparent stretchable conductors. The electrical properties of these conductive composites are also dependent on dispersion techniques [48] and properties of matrix [77,119].

The blended conductive composites can be further patterned or used as conductive inks. Photo-patternable conductive PDMS was fabricated by blending in both conductive fillers and photosensitizers [146]. A composite ink of soluble silver salt and adhesive rubber was directly filled into ballpoint pens to write on different substrate to form adhesive conductive tracks [147]. Ag/PDMS composite ink was stencil printed and screen printed onto PDMS substrate to fabricate soft printed circuit boards (PCBs) [19].

### 3.2. Conductors in Microchannels

Filling the PDMS microchannels with liquid metals is another straightforward method of fabricating stretchable conductors. The PDMS microchannels are typically fabricated by molding top and bottom layers from the SU-8 master and adhering these two layers by oxygen plasma treat, heat, and pressure [144,148]. In the initial stages, solder was melted and filled into PDMS channels to fabricate stretchable conductors [149]. This process needed heating (up to 180 °C) and cooling steps, which was hence incompatible with heat-sensitive materials [141]. Filling liquid metals into the microchannels circumvents the heating during fabrication. The liquid nature endows them with excellent stretchability and self-healability [140]. Conductors fabricated by filling diamond-shape PDMS microchannels with EGaIn were stretched biaxially to 100% strain [144] while retaining their characteristics. Wetting of PDMS with the liquid metal was an issue which was solved effectively by putting on a metal layer [144] or applying silane adhesion promoter [149]. This strategy was also exploited to strengthen the brittle areas, such as the sharp corners of the diamond-shaped metallic conductive tracks [150].

### 3.3. Stretchable Network on/Embedded in Elastomers

As discussed in Section 2, the nanomaterials with high aspect ratio are typically arranged into networks possessing stretchability. Such network films are ready to be fabricated onto the elastomeric substrate without applying any other mechanics. Some nanomaterials can be directly solution- processed onto the modified elastomeric substrate [112]. For example, the self-assembly of functionalized SWCNTs onto PDMS substrate was reported [151]. Besides the direct fabrication of network films onto elastomer, transferring a solid film to the elastomeric substrate by conformal contact (due to the good adhesion between PDMS substrate and the conductive network film), or using the transfer media [27,41], provides alternative routes to obtain conductive network films on the elastomer surface [42,43]. In addition to these methods, vacuum suction is also an effective method to transfer conductive network film from membrane filter to elastomeric substrate [40,75,122].

**Figure 4:**
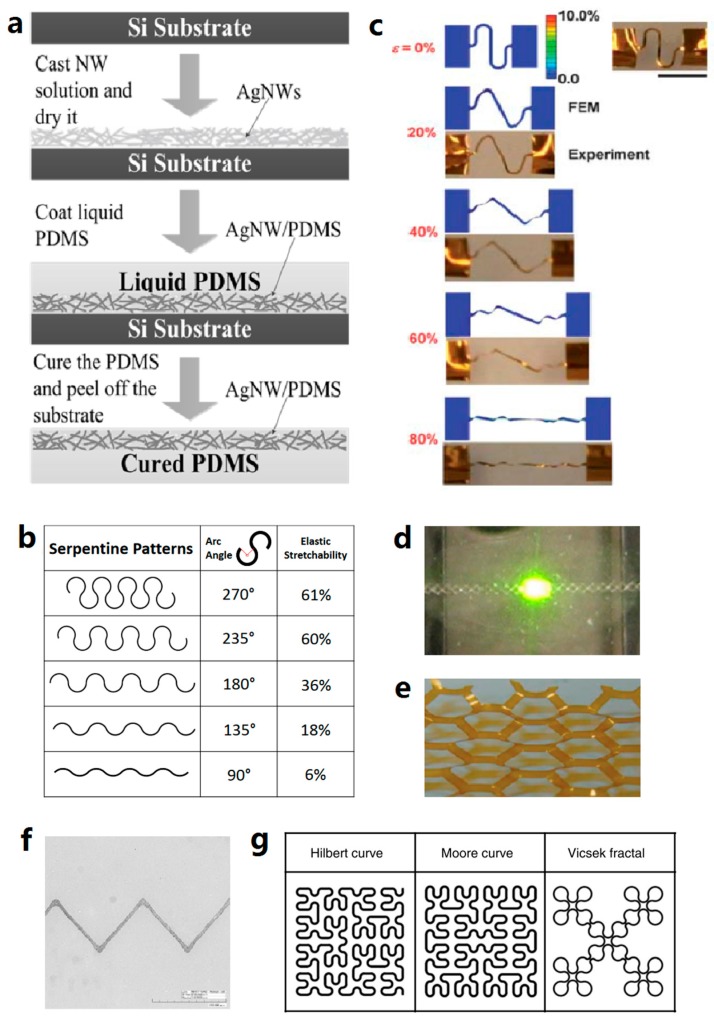
(**a**) Schematic of the fabrication process of an AgNW/PDMS stretchable conductor. Reprinted with permission from [21]. Copyright 2014 American Chemical Society; (**b**) Simulated uniaxial elastic stretchability for horseshoe patterns as a function of height/spacing ratio (arc angle). Reprinted by permission from Macmillan Publishers Ltd.: *Nat. Commun.* [102], Copyright 2014; (**c**) Experiments and FEM analyses on the symmetric buckling behavior of the serpentine interconnect with strain from 0% to 80% (scale bar: 1 mm). Reproduced from [156] with permission of The Royal Society of Chemistry; (**d**) Diamond-shaped stretchable interconnects based on liquid metal. Reprinted from [144], with the permission of AIP Publishing; (**e**) Honeycomb-patterned polyimide substrate at stretched state. Reprinted with permission from [153]. Copyright 2011 American Chemical Society; (**f**) Zigzag Cu conductive tracks on PDMS [100]. Reproduced with permission from ^©^ IOP Publishing. All rights reserved; (**g**) Three representative fractal patterns. Reprinted by permission from Macmillan Publishers Ltd.: *Nat. Commun.* [102], Copyright 2014.

The nanomaterial-based conductive networks can be embedded into the elastomer as well. The conductive networks are fabricated on a donor substrate. The conductive composites are obtained by coating the liquid elastomer precursor onto the donor substrate (thus the precursor infiltrates into the networks), curing and peeling off [82,104]. This method is also suitable for fabricating conductive networks with relatively large thickness, such as the vertically aligned CNT forests [45,76,123]. Figure 4a gives a schematic of such fabrication process of an AgNW/PDMS stretchable conductor.

### 3.4. Geometry Design

Designing the geometry of conductive tracks to reduce the strain in the metals, is an intelligent strategy in the fabrication of metallic film-based stretchable conductors. The realization of the geometry largely relies upon the patterning techniques, which are discussed in Section 4.

Here, we categorize those geometries which endow stretchability to the conductors into three types: polygonous, serpentine, and fractal designs. The polygonous designs make metallic tracks into connected polygonous networks, such as diamond [150,152] and hexagon (honeycomb) [153]. Serpentine designs include zigzag [100], half circle (U-shape) [101,154], sinuous [6,13], and horseshoe [100,101,155]. Among them, the horseshoe design provided the best stretchability [101]. Parameters such as the linewidth of metal, thickness of metal, and height/spacing ratio of geometry in the serpentine design play an important role in the performance of these conductive tracks. It was proven experimentally [13] and with the finite element method (FEM) analyses [101] that subdividing a wide conductive line into several thinner lines largely improved the stretchability and alleviated the stretching-induced stress. Analysis of postbuckling of the serpentine interconnects revealed that the elastic stretchability of the serpentine interconnects increased with decreased thickness [154,156]. The height/spacing ratio of geometry refers to the ratio of height (perpendicular to the stretching direction) to spacing width (parallel to the stretching direction). Increased height/spacing aspect ratio benefits the stretchcability when it is under a certain range [102,154]. In Figure 4b, the simulated uniaxial stretchability of the horseshoe patterns, with varied height/spacing ratio (arc length), is given [102]. As shown in the figure, the stretchability keeps increasing until the arc angle reaches 235°. Fractal designs can be perceived as the assembly of serpentine designs, which enable unusual mechanics with implications in stretchable device design [102]. In Figure 4c, the stretchability of a freestanding U-shape serpentine copper interconnect in symmetric buckling mode is demonstrated both experimentally and with FEM analyses. Some other representative geometry designs are shown in Figure 4d–g.

### 3.5. Buckling

The buckling phenomenon was first found in the metal thin film on PDMS. Due to the large thermal expansion of PDMS, the subsequent cooling processes caused compressive stress in the metal film, which was calculated using Equation (2).

(2) $$\sigma_{0}=\frac{E_{m}\left(\alpha_{p}-\alpha_{m}\right)\left(T_{D}-T\right)}{\left(1-\nu_{m}\right)}$$

where σ_0_ was the compressive stress in the metal film. *E*, α and *ν* were referred to as Young’s modulus, thermal expansion coefficient, and Poisson’s ratio, respectively. The subscripts *m* and *p* referred to metal film and PDMS, respectively. *T* and *T_D_* were the real-time temperature and the deposition temperature [1]. The compressive stress developed in the metal film led to buckling. It is worth mentioning that an interfacial adhesion layer of titanium or chromium was critical in making metal film compliant to PDMS. The buckling of metal film on a flat, unconstrained PDMS was however, uncontrollable. Ordered buckling was obtained by molding the PDMS surface with certain patterns or by partial modification of the surface [72].

The idea to utilize the pre-stretched elastomer to get buckled metal film further propelled the application of buckling strategy in stretchable conductors. In this approach, metal film was evaporated on 15% pre-stretched PDMS with an adhesion layer and patterned by standard lift-off process. Upon releasing the PDMS from the fixture, the metal film stayed compliant on the surface of PDMS and buckled. The as-prepared metal film was stretched, with stable resistance up to 25% [92]. Analytical models were proposed to predict the wavelength and amplitude of the buckling [39,70,157,158]. Interested readers can refer to a review on mechanical buckling [159], where the buckling phenomenon was studied in cases of compliant/small strain, compliant/large strain, and delaminating.

**Figure 5:**
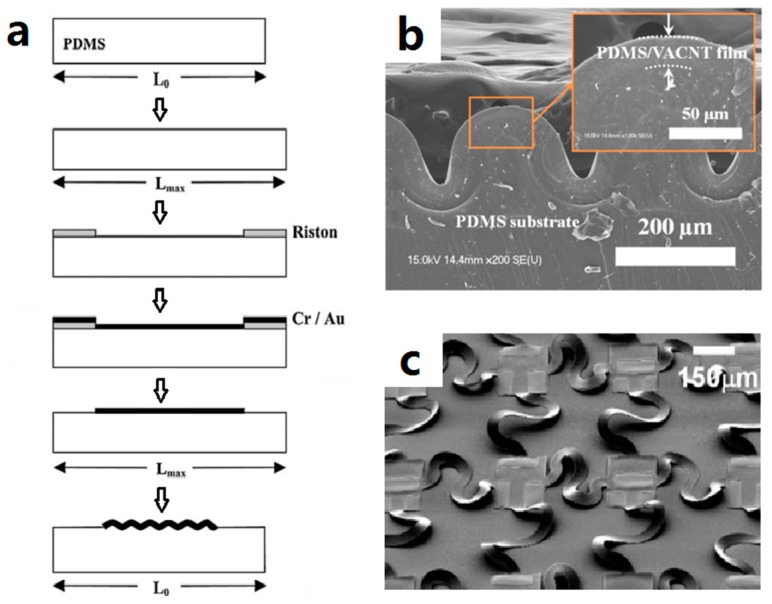
(**a**) The schematic of the fabrication of Au interconnects on PDMS substrate with buckling strategy. ^©^ 2004 IEEE. Reprinted, with permission, from [92]; (**b**) Cross-sectional SEM image of a VACNT/PDMS conductive film. The inset shows the magnified SEM image of the composite part. Reprinted with permission from [45]. Copyright 2014 American Chemical Society; (**c**) SEM image of an array of stretchable complementary metal-oxide-semiconductor (CMOS) inverters with non-coplanar bridges [160]. Copyright 2008 National Academy of Science.

With the advents of more and more electrical conductive materials in stretchable conductors, the buckling strategy was explored in stretchable conductors based on CNTs [42,57], graphene [128], AgNWs [113], conductive polymers [133], and a combination of other interesting mechanics [160]. Figure 5b shows the SEM image of the buckling of an elastomer-infiltrated vertically aligned carbon nanotube (VACNT) film. This film was reported as having a very small resistance change (ratio of less than 6%) when stretched to the level of pre-strain (100%).

Buckling was also reported without the pre-strain of substrate. The buckling of AgNW/PDMS film emerged upon the first stretch/release cycle. Such films could maintain stable conductivity of 5285 S·cm^−1^ at the strain range of 0%–50% [104]. A similar strategy was employed to fabricate laterally buckled CNT ribbons on PDMS [43].

### 3.6. Out-of-Plane Design

According to some studies of stretchable conductors, the conductive materials were supposed to have good adhesion to the substrate [10,161]. However, it was also pointed out that, for serpentine metallic lines on elastomer, the lines would deform out of plane of the substrate to alleviate the strain [101]. The strong bonding with the substrate would constrain such deformation and thus increase the plastic strain and in-plane shear strain [155].

An innovative approach to fabricate stretchable conductors is to make them free of substrate constraints. Figure 5c shows an array of stretchable complementary metal-oxide-semiconductor (CMOS) inverters with non-coplanar bridges. The islands with devices are well-attached to the substrate, while the interconnecting bridges are lifted off from the substrate to accommodate deformation [160]. Similar structures which contained anchored square islands and S-shaped suspensions were also reported [162].

### 3.7. Designs in Substrate

Generally, electrical conductors of a stretchable device are prone to failure. That is why most of the mechanics of strechability are focused on the electrical conductors. However, designing the substrate is also a good strategy to broaden the mechanics of stretchable electronics. One way to enhance stretchability of the substrate is to use two substrates with different Young’s modulus so that the substrate with a higher modulus can sustain lesser strain [52,53]. For example, in a stretchable device array, stiff PDMS islands were used for device assembly, while soft Ecoflex substrate was designed to sustain the deformation. PDMS relief, consisting of raised islands and recessed trenches, was used as the substrate for rigid GaAs photovoltaics. It was found that stretching the substrate to an overall strain of 20% induced 123% strain in the trenches and only 0.4% strain at the islands [54]. Fabricating the substrate into a wavy shape would largely improve the stretchability of the whole system [24,125,160,163]. Besides that, a novel shape-memory shrinkable polymeric substrate utilized in the fabrication of highly stretchable gold films was reported [164].

## 4. Patterning Techniques

Patterning is one of the key techniques in the fabrication of both wafer-based electronics and stretchable conductors. The common patterning techniques for stretchable conductors are lithography, screen/stencil printing, direct printing, and transfer printing. The techniques from conventional wafer-based electronics, such as photolithography and lift-off, are subsequently adopted in stretchable electronics [17]. Besides, stencil printing and screen printing are employed to pattern features that do not require high complexity and superfine resolution [19,20,21]. With the development of additive manufacturing, direct printing emerges as an alternative to the conventional subtractive patterning method [14,22,23,24,25,26,27,28,29,30,31]. Transfer printing generally means to transfer functional materials from one substrate to another. With a mold-patterned elastomer stamp, those materials can be selectively transferred from donor substrate to the target substrate [32,33,34].

### 4.1. Lithography

Subtractive patterning techniques, such as photolithography and lift-off, are well-developed and dominatingly used in wafer-based electronics. However, in elastomer-based stretchable conductors, the elastomers are mostly incompatible with the chemicals and high energy beams used in these processes. Despite that, the patterning of devices still relies on lithography, where high resolution and complexity is required. In addition to the photo-defining and etching steps, an extra transfer step is needed. The patterning of the conductive materials is initially processed on regular substrates such as silicon wafer or glass and then transferred to elastomeric substrate. The transfer step can be done by directly coating the liquid elastomer precursor, curing, and peeling off (similar to the process shown in Figure 4a) [165]. It is also achievable by picking up and releasing the conducive materials with the help of an intermediate substrate [102,166,167].

### 4.2. Stencil/Screen Printing

**Figure 6:**
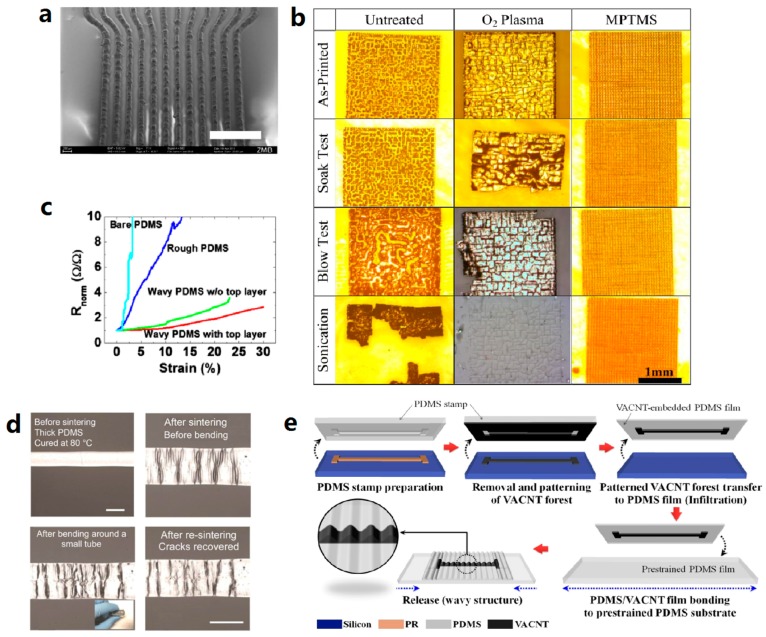
(**a**) SEM image of stencil printed Ag/PDMS tracks with the highest resolution achieved (scale bar: 1 mm). Reprinted by permission from Macmillan Publishers Ltd.: *Sci. Rep.* [19], Copyright 2014; (**b**) Photographs from adhesion tests of untreated, O_2_-plasma treated, and adhesion-promoter (MPTMS)-treated 2 mm square ink-jet-printed Ag patterns on PDMS under destructive tests. ^©^ 2014 IEEE. Reprinted, with permission, from [69]; (**c**) Changes in resistance as a function of strain of the ink-jet-printed silver tracks on PDMS substrate with varied adhesion situations. Reprinted from [24], with the permission of AIP Publishing; (**d**) Morphologies of ink-jet-printed silver track on PDMS before and after sintering, after bending, and after re-sintering (scale bar: 100 μm) [26]. ^©^ IOP Publishing. Reproduced with permission. All rights reserved; (**e**) The schematic of fabrication process of PDMS/VACNT-film-based wavy-configured stretchable conductors. Reprinted with permission from [45]. Copyright 2014 American Chemical Society.

Compared to the lithographic approach, direct deposition of conductive materials through a mask, stencil, or screen onto the substrate is relatively simple and cost-effective. It is suitable for applications that do not require high complexity and superfine resolution. Figure 6a is an SEM image of stencil-printed Ag/PDMS composite with the highest resolution achieved (linewidth of 150 μm with spacing of 100 μm).

This approach is versatilely utilized with various conductive materials. Gold strip on PDMS was made by evaporating gold on PDMS laminated with patterned Dupont Riston photoresist film [92]. Liquid metals embedded into elastomer were fabricated using masked deposition [168]. A strain sensor array was fabricated by cross-folding screen printing AgNW stripes on PDMS [20]. Ag/PDMS composite was both stencil printed and screen printed to obtain soft PCBs [19]. PEDOT:PSS ink was stencil printed on Ecoflex to fabricate electrochemical sensors [51].

### 4.3. Direct Printing

Owing to the development of additive manufacturing, conductive inks can be directly printed on various substrates without physical contact. Various printing techniques, including laser-aided direct writing [22], ink-jet printing [23,24,25,26], and aerosol-jet printing [14,27,28,29,30] have been developed for printed electronics. The maskless processes are well suited for rapid prototyping. Also, they offer a cost-effective way to achieve large-area electronics production with minimal materials waste and without lengthy subtractive processes. Both planar and 3D architectures are achievable by laser-aided direct writing [169]. Ink-jet printing is a well-established technique for graphical printing and has been extensively used for printed electronics [170]. In this technique, the ink drop is driven by thermal, piezoelectric, or electrostatic actuation and delivered through a printing orifice to the substrate at a demanded area [171]. Aerosol-jet printing is a relatively new technique. Instead of printing the ink drop by drop, it prints the aerosol mist generated by ultrasonic or pneumatic atomizer. Aerosol-jet print has the capability of generating smaller feature size [30], as compared to the ink-jet printing and is compatible with 3D non-planar substrates [170]. The broad applications of such direct printing methods have been demonstrated by printing various conductive materials, such as metallic NP inks [24,69], CNTs [28,30,172], conductive polymers [27,173,174], and dielectric materials, such as ion gel [27,174] and polyimide [28], on not only regular flexible plastic substrate but also elastomeric substrates.

One of the issues in the direct printing technique is that of wetting and adhesion between elastomers and conductive inks. PDMS is known to have surface hydrophobicity, which can be modified to hydrophilicity by O_2_ plasma [68,69], UV/ozone [24,70,71], and chemical treatment [68]. The surface treatment of PDMS imparts good wetting of PDMS with various inks and promotes the adhesion between PDMS and conductive materials. However, in some cases, such adhesion might not be strong enough and require further deposition of adhesion layer or blending of an adhesion promoter in the initial stages. Super-thin (5 nm) titanium or chromium adhesion layer was largely used to bond gold thin film with PDMS [1,12,92,162,175]. Poly-dopamine adhesion layer was employed to significantly modify the surface property of PDMS and enhance the adhesion between PDMS and spray-deposited AgNWs [112]. Zonyl, as a non-ionic fluorosurfactant, can be mixed in both elastomer precursors and conductive inks, which enhances the interfacial adhesion between elastomers and various conductive materials [7,51,71,176]. Silane adhesion promoter MPTMS (3-mercaptopropyl trimethoxysilane) is widely used in PDMS-based stretchable conductors. It was used as the adhesion promoter between PDMS and molten liquid solder [149], evaporated gold film [165,177], and printed metallic inks as well [25,69]. In Figure 6b, the adhesion tests of the ink-jet- printed silver patterns on PDMS with blank treatment, O_2_ plasma treatment, and MPTMS treatment are compared. Only the MPTMS-treated sample survived the three destructive tests. [69]. The influence of the adhesion on the stretchability of ink-jet-printed silver tracks is demonstrated in Figure 6c. The PDMS rough surface obtained from UV/ozone treatment provided better adhesion, thus making the tracks more stretchable. Besides that, a top PDMS layer encapsulated the silver tracks, making them compliant to the wavy PDMS and hence further enhancing the stretchability [24].

Besides the adhesion issue, many other problems, such as the swelling [62], thermal hardening, and large thermal expansion of the elastomers, still exist in the direct printing of conductive inks [26], resulting in poor performance of printed stretchable conductors. Cracking [26,31] and low stretchability are some of the resulting issues found in printed stretchable conductors [14]. As an example, Figure 6d shows the morphologies of ink-jet-printed silver track on PDMS before and after sintering, after bending, and after re-sintering [26], in which the silver track cracked as a result of the thermal expansion problem. All these problems and issues account for the current situation that most of the stretchable conductors are not directly fabricated on elastomers. This should be addressed in order to realize the direct printing of stretchable conductors.

### 4.4. Transfer Printing

The challenges involved in direct printing of stretchable conductors are discussed above. Transfer printing is an alternative approach, which effectively circumvents such difficulties. In a general sense, transfer means switching the substrate of the conductive patterns. As mentioned in Section 4.1, coating the liquid elastomer precursor onto the donor substrate, curing, and peeling off is a typical transfer process [82,104,165].

Transfer is also achievable via intermediate media. Poly(methyl methacrylate) (PMMA) is one of the common transfer media [46,132]. Conductive materials can be embedded into PMMA like in PDMS and transferred to the target substrate, followed by dissolving PMMA in hot acetone. Some other materials, such as functional tapes, were also reported as the transfer media. Water-soluble tape is an adhesive tape which is dissolvable in water. Conductive materials on donor substrate could be archived by using such tape and releasing them on the target substrate. The tape would totally disappear when dipped in water, leaving only conductive materials on the target substrate [27,41,56]. Thermal release tape is an adhesive tape which loses its adhesion upon heating. Thermal release tape was reported as a temporary holder to transfer NW-based devices [178]. Besides the transfer media, a sacrificial layer is an essential assistant in transfer printing techniques. A sacrificial layer is typically fabricated as a structural support between the donor substrate and the conductive materials. This layer can be removed by solvent or etchant, thus lifting off the conductive materials and making them easily transferable. The commonly used sacrificial materials include copper [101], gold [177,179], PMMA [160], SiO_2_ [46,157], and hard-baked photoresist [164,180].

Transfer by PDMS stamp is extensively used in soft lithography, where the conductive materials can be selectively transferred from donor substrate to the target substrate using a mold-patterned PDMS stamp. Transfer printing by such PDMS stamps is either additive or subtractive [32]. The key to the PDMS stamp-based transfer process is controlling the adhesion. The adhesion of the PDMS stamp to the donor or the target substrate can be controlled kinetically, owing to the rate-independent viscoelastic property of PDMS [33]. The adhesion can also be controlled by manipulating the surface chemistry and the interfacial properties of the PDMS stamp. [34].

As an example of transfer printing, Figure 6e illustrates the fabrication of stretchable vertical-aligned carbon nanotube (VACNT) film on PDMS substrate, in which two transfer processes were involved. A subtractive transfer printing technique was used to remove the undesired part of VACNT film. The remaining part was transferred by an intermediate PDMS substrate to a pre-stretched target PDMS substrate.

## 5. Applicable Devices

Stretchable electronics have been developed into a unique and emerging field of electronics. They contravene the common idea that people would attach to electronics—stiff circuit board and cold metals. They make electronics closer to human. Because of the soft, attachable nature, stretchable electronics show great potential in developing strong intimacy with humans, like conforming to the skin (epidermal electronics) [18,35] and implanting into the body [36,37]. They have an enormous potential in future electronics for biomedical use, such as lightweight physiological monitors and in personalized healthcare [60,61]. Stretchable conductors are the most essential building block of stretchable electronic devices. They could simply serve as interconnects, or as functional electrodes. In this section, the applications of the stretchable conductors are discussed by demonstrating the functional devices based on them.

### 5.1. Sensors

Stretchable sensors are probably the most widely studied type of devices in the recent development of stretchable electronics. Stretchable sensors with various functions have been fabricated, including strain sensors [181], pressure sensors [20,59,107,182], temperature sensors [18,183], gas sensors [132,184], and UV sensors [53]. These sensors detect the changes in the outside environment and reflect these changes in the electrical properties, such as resistance and capacitance. Sensors are also fabricated based on the performance of devices such as transistors [152] and antennas [21].

**Figure 7:**
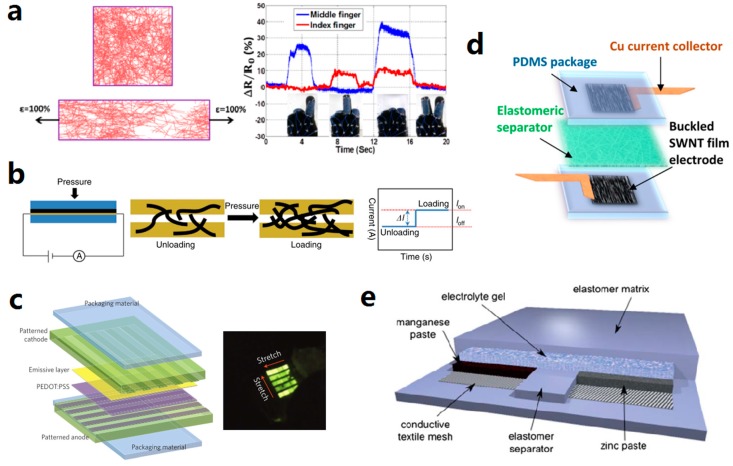
(**a**) Schematic of AgNW network at relaxed and stretched states and the illustration of the motion detect function of an AgNW/PDMS strain sensor. Reprinted with permission from [111]. Copyright 2014 American Chemical Society; (**b**) Schematic of the sensing mechanism of an AuNW-based pressure sensor. Reprinted by permission from Macmillan Publishers Ltd.: *Nat. Commun.* [107], Copyright 2014; (**c**) Schematic and photograph of an encapsulated fully stretchable elastomeric polymer light-emitting devices (EPLED) display with 25 pixels. Reprinted by permission from Macmillan Publishers Ltd.: *Nat. Photonics* [185], Copyright 2013; (**d**) Schematic of the components of a dynamic SWCNT (Single-wall carbon nanotubes)-based stretchable supercapacitor. Reprinted with permission from [57]. Copyright 2012 American Chemical Society; (**e**) Schematic of a rechargeable stretchable alkaline manganese cell. Reproduced from [83] with permission of The Royal Society of Chemistry.

Stretchable strain sensors have largely extended the sensing range beyond those of traditional non-stretchable strain sensors from below 1% [136] to several hundred percent [181]. The strain sensors are mostly resistive or capacitive. Stretchable conductors with reversible and repeatable resistance change under strain are suitable for fabricating resistive strain sensors. Carbon-based materials are widely used in this application due to their intrinsic piezoresistivity [46,47]. Network structures fabricated from AgNWs [111] or CNTs [41] are also piezoresistive, with mechanisms of local disconnections and tunneling effects [181]. In capacitive strain sensors, an elastomeric dielectric layer is sandwiched between two stretchable electrodes. At certain strain, the decreased width and thickness of the dielectric layer results in changes of capacitance. Both AgNWs and CNTs were reported as the electrode materials [20,59,80]. Figure 7a shows a resistive stretchable strain sensor based on AgNW/PDMS nanocomposites. More information on the stretchable strain sensors can be found in a recent review [181].

Stretchable pressure sensors detect the vertical deformation caused by pressure and send out differential electrical signals. Capacitive pressure sensors have a similar mechanism to capacitive strain sensors. When the capacitor is under pressure, the distance between the two electrodes decreases, causing changes in capacitance. Capacitive sensing arrays based on AgNWs and various elastomers have been demonstrated with the functionality as strain sensor, pressure sensor, and touch sensor [20,59]. A stretchable resistive pressure sensor was fabricated by sandwiching AuNW coated tissue sheet with an interdigitated electrode between two PDMS sheets, as shown in Figure 7b. The applied pressure was determined by measuring the changes in resistance caused by the different loading of AuNWs on the electrode [107].

Noble metals, such as gold and platinum, are resistance-sensitive under varied temperature. Both traditional temperature sensors and stretchable temperature sensors take advantage of this property. However, these noble metals are not intrinsically stretchable. To incorporate them into stretchable temperature sensors, mechanics to enhance their stretchability were employed. Platinum meander electrodes were employed as temperature sensors in an integrated epidermal electronic system [18]. The buckling strategy was used to fabricate stretchable temperature sensors by transferring gold strips onto pre-stretched PDMS [183].

In most of these stretchable sensors, rough structures, such as NW networks and buckled films/strips, are well encapsulated or packaged with elastomers [20,59,183]. However, the influence of such rough structures on the device performance has not yet been thoroughly discussed, and this is expected to be addressed in the near future.

### 5.2. Light-Emitting Circuits

Light-emitting circuits on elastomeric substrates are designed to meet the demands of flexible, wearable, and foldable displays and light sources. In these circuits, stretchable conductors serve as interconnects between the power supplies and the light-emitting devices. EGaIn liquid metals embedded into soft elastomeric substrate were used as interconnects to light-emitting diode (LED) arrays on stiff elastomeric islands [53]. A stretchable heart-shaped light-emitting circuit was fabricated by installing LED lights onto hand-written stretchable tracks from an adhesive silver-based conductive ink [147]. In addition, serpentine graphene interconnect bridges were also used as interconnects for microscale inorganic LED array [129].

Besides interconnects, stretchable conductors were used as electrodes in thin film polymer light-emitting devices as well. Liang et al. demonstrated an elastomeric polymer light-emitting devices (EPLED) fabricated by sandwiching an emissive polymer layer in AgNW-PU composite electrodes. Such a structure was further developed into a stretchable EPLED display array of 25 pixels. The schematic and photograph of the display array are shown in Figure 7c [185].

### 5.3. Transistors

Stretchable conductors are utilized in stretchable transistors as interconnects and electrodes for gate, source, and drain. An SWCNT-based elastic conductor was used to form interconnections between contact pads of an organic transistor-based active matrix [121]. Polymer-sorted and unsorted CNTs were used as the semiconductor and conductor, respectively, in a thermoplastic polyurethane (TPU)-based stretchable transistor [186]. Graphene/AgNW hybrid conductors were used as the drain/source electrodes of an oxide semiconductor transistor array [187]. A stretchable and transparent transistor array was fabricated with patterned graphene thin film as the semiconducting channel and source/drain electrodes. The patterned graphene was transferred to the rubber substrate, followed by aerosol printing of ion gel and conductive polymer as the gate dielectric and gate electrode, respectively. Such graphene transistors showed hole and electron mobilities of 1188 ± 136 and 422 ± 52 cm^2^/(V·s), respectively, with stable operation at strain up to 5%, even after more than 1000 cycles [27]. Highly stretchable polymer transistors made entirely of stretchable components were fabricated on SBS fiber mat with stacked Au nanosheets as electrodes and poly(3-hexylthiophene) (P3HT) nanofibers as channel material. The transistors provided a hole mobility of 18 cm^2^/(V·s) at a strain of 70% [188]. Recently, an intrinsically stretchable organic thin film transistor was reported with a healable polymeric semiconductor and CNT/PEDOT:PSS electrodes. Such a transistor was able to sustain a field-effect mobility of 1.12 cm^2^/(V·s) at a large strain of 100% [189].

### 5.4. Energy Devices

Almost all of the stretchable electronic devices with amazing functionality were connected to and driven by external power sources. Low cost and compatible internal power supplies for independent stretchable electronic systems are in great demand, as they can facilitate the real-life usages of these stretchable devices. Energy devices are basically divided into energy storage devices and energy conversion devices. Although many research works focused on stretchable energy devices such as supercapacitors [57,58,125,132], batteries [83,84,134,166], solar cells [176], and other environmental energy harvests, challenges still remain to fabricate stretchable energy devices with high performance and reliability. Here, in this part of the review, these devices are briefly introduced with an emphasis on the usage of stretchable conductors. Two recent review papers about stretchable energy storage and conversion devices can be referred to for further information [38,190].

Supercapacitors are commonly used as energy storage devices. Carbon-based nanomaterials are ideal for this application due to their good stability, conductivity, and high surface area. Buckled SWCNT films on PDMS substrate as electrode, together with organic electrolyte and a separator layer were used to fabricate stretchable supercapacitors [57,58]. The schematic of the components of an SWCNT-based supercapacitor is shown in Figure 7d. In addition to this, highly-aligned CNT sheets were fabricated from vertically-grown CNT forest and further used in an all-solid supercapacitor [191]. Micro-supercapacitor array based on P3Ain-wrapped MWCNTs was employed to drive stretchable graphene gas sensors [132]. Graphene microribbons were also utilized as the electrode materials in stretchable supercapacitors [125].

Unlike supercapacitors, batteries store energy in forms of chemical energy. Stretchable batteries of various structures and shapes have been fabricated with several materials till now. Buckled polypyrrole macrofilm cathodes on elastomeric substrate were reported for battery applications [134]. Porous CNT/PDMS nanocomposites fabricated by phase separation were used as the anode in a flexible lithium-ion battery [192]. Intrinsically stretchable and rechargeable cells were fabricated by embedding chemically reactive pastes (zinc anode and manganese dioxide cathode) and electrolyte gels into an elastomer matrix, as shown in Figure 7e [83,84]. Although the abovementioned batteries could easily provide energy by electro-chemical process, they still needed to get recharged by physical contact with external power sources. Xu et al. demonstrated stretchable battery arrays with an integrated wireless recharging system. The wireless recharging system, consisting of wireless coil, Schottky diode, and parallel capacitor, was designed to recharge these batteries without direct physical contact [166].

Energy harvesters exploit environmental energy and generate power for the stretchable devices. Solar cells are one of the well-developed types of energy harvesters. To fabricate stretchable solar cells, various stretching mechanics were exploited to incorporate the stiff photovoltaic materials. Stretchable organic solar cells were fabricated by depositing a PEDOT:PSS electrode, organic photovoltaic materials, and EGaIn as the top contact, sequentially on a pre-stretched PDMS substrate. The 3D wavy buckles appeared in the film upon releasing and imparted the solar cells with reversible stretchability [176,193]. Stretchable dye-sensitized solar cells were fabricated using elastic conductive fiber by spinning MWCNT sheets on rubber fiber. Modified titanium wire was wound onto the fiber as the working electrode, followed by coating with photoactive materials. The devices had open-circuit voltages of 0.71 V and energy conversion efficiency of 7.13% [194]. Besides the solar cell, other energy harvesters that have potential but have not yet been well-studied in stretchable electronics include piezoelectric motion energy harvesters [195,196], RF energy harvesters [60,197,198], the thermal energy harvester [199], and triboelectric energy harvester [200].

## 6. Summary and Outlook

In this review, we went through the materials, mechanics, and patterning techniques of stretchable conductors and the applicable devices. Stretchable conductors are the basic building block of stretchable electronic devices. Their compliant, deformable virtues have changed the common idea that people would have on rigid silicon-based electronics and opened up a new direction of the next generation of electronics. They have enormous potential in biomedical engineering, which has been demonstrated in many research works [4,18,60,61,94,167,181,201]. Besides that, they have application in broad fields such as wearable communication devices [202], prosthetic electronic skins [203], soft robotics [204], and interactive human–machine interfaces [205]. We foresee a bright future for stretchable electronics as they bring electronics closer to humans.

Despite the huge advancements in stretchable electronics so far, there still exist some issues and challenges that should be solved in order to realize the use of stretchable electronics in everyday life. First, the long-term stable performance of these stretchable electronics, which would be influenced by the stability of conductors and the aging of substrates [206,207,208], has not yet been demonstrated comprehensively. Second, as many stretchable electronics are aimed at biomedical applications, their biocompatibility should be examined extensively. Although the biocompatibility of some materials has been demonstrated [209], however, thermal management of the stretchable devices, where the heat generated may cause tissue lesioning, are relatively less studied [210,211]. Third, the large mismatches in Young’s modulus, elongation at break, and thermal expansion of various materials used in stretchable electronics have posed great challenges in integration with the systems. Several stretching mechanics have been proposed which effectively remedy these integration challenges. However, problems such as cracking and delamination are still reported when the system reaches a certain strain. More fundamental understanding of the causes of failure and interfacial properties between heterogeneous materials is needed in order to achieve better performance of novel stretchable devices. Fourth, some of the stretching mechanisms and structures are hard to implement in large-scale manufacturing, such as pre-strain-induced buckling and out-of-plane design. Efforts are still required to figure out cost-effective, scalable fabrication routes with high reliability, repeatability, and precision for the purpose of commercialization. For stretchable electronics based on nanomaterials, repeatability of the device’s performance from batch to batch largely relies on the uniformity of the nanomaterials, which is also challenging in itself. Lastly, despite stretchable electronics with manifold functional devices having been demonstrated, integrated internal power supplies are still in great demand for self-powering stretchable electronic systems.

Based on the current trend, the possible future directions of stretchable electronics are foreseeable. First, the diversity of materials used would continuously expand. The rate of discovery and development of new materials are beyond our expectations. Take graphene as an example, it only took about five years from its first discovery [212] to its first utilization in stretchable conductors [118]. For sure, more and more organic, inorganic, conducting, semiconducting, and dielectric materials would provide opportunities in stretchable electronics. As for the substrate and matrix, besides the widely used PDMS, there are emerging elastomeric materials such as rubber fiber mats. Second, the design of discrete devices and their integration in stretchable systems would grow quickly. Stretchable electronics with sensing functions have gained considerable progresses. However, devices with other functions such as memory [213] and communication [202,214] are still in infancy. There is also an urgent demand for energy devices, and many research groups are working to address this issue. Third, with the increase of complexity and resolution of devices, higher requirements for patterning techniques are expected. Direct printing, as an additive manufacturing method, would satisfy such requirement and offer low cost and high speed in both prototyping and manufacturing. It might be a solution for cost-effective and scalable fabrication of stretchable electronics.

## Figures and Tables

**Table 1:** Elastomers for stretchable conductors and their elastic properties.

Material	Tensile Strength (MPa)	Maximum Strain (%)	Young’s Modulus (MPa)	Reference
PDMS	6.25	120–160	2.05 (strain < 40%)	[52,55]
Ecoflex^®^ 00-30	1.38	900	0.07 (strain = 100%)	[87]
Polyurethane	7.32	760	7.82 (initial)	[76]
IIR	3.51	170	0.41 (strain < 40%)	[55]
POE fiber mat	-	>600	-	[78]
SBS fiber mat	-	>530	0.47 (low strain)	[79]
Dragon Skin^®^ 30	3.45	364	0.59 (strain = 100%)	[88]
Solaris	1.24	290	0.17 (strain = 100%)	[89]
Acrylic elastomer (VHB 4910)	0.69	-	-	[90]
NBR	10	250	-	[91]

**Table 2:** Conductive fillers for stretchable conductors based on blending.

Material	Size	Percolation Threshold (wt %)	Reference
Silver particles	1–2 μm	83	[50]
Carbon black	40–100 nm	10	[50]
MWCNT	Diameter: 60–100 nm	2	[48]
	Length: 5–15 μm		
PEDOT	-	23	[49]
